# On-Site Sensitive Colorimetry for Free Cyanide by Using Ion-Pair Solid-Phase Extraction with Sedimentable Dispersed Particulates and Mobile Photography Box

**DOI:** 10.3390/molecules29225371

**Published:** 2024-11-14

**Authors:** Nozomi Kohama, Takuya Okazaki, Kazuto Sazawa, Noriko Hata, Hideki Kuramitz, Shigeru Taguchi

**Affiliations:** 1 Department of Natural and Environmental Sciences, Faculty of Science, Academic Assembly, University of Toyama, Toyama 930-8555, Japan; 2 Department of Applied Chemistry, School of Science and Technology, Meiji University, Kanagawa 214-8571, Japan

**Keywords:** solid phase extraction, particles, cyanide, mobile photography box

## Abstract

We propose a sensitive and simple colorimetric method using dispersed particle extraction for the highly sensitive detection of free cyanide in water samples. The method involves the direct capture of the color-producing compound by dispersed microparticles in a sample vessel containing colorimetric reagents and an adsorbent. The color of the microparticles that have naturally settled to the bottom of the microtube can be directly measured by visual observation or image analysis. A mobile photography box (MPB) suitable for use with a smartphone was developed to ensure reproducibility in the lighting environment during image acquisition. The MPB was then used to develop a highly sensitive analytical method for cyanide. This newly developed method allows direct measurement of the color tone of the target component collected on particles by irradiating light from below and photographing it perpendicularly. The method offers excellent portability, sensitivity, and reproducibility and is less affected by interfering color components. When applied to cyanide analysis, the detection limit reached 0.005 mg/L and measurements could be completed within 10 min, significantly shorter than the conventional absorbance spectrophotometric method, which requires 30 min. Thus, this method achieves highly sensitive cyanide analysis.

## 1. Introduction

Cyanide contamination of surface water sources and soils due to industrial activities such as gold mining, electroplating, municipal waste burning, and pesticide production is a serious environmental concern [1,2]. Among cyanides, free cyanide (CN^−^) [3], which exists as cyanide ions and hydrogen cyanide, easily forms complexes with metals and is highly toxic even at low concentrations. Even trace quantities of CN^−^ can have a detrimental impact on aquatic organisms and microorganisms when dispersed in the environment. International organizations such as the World Health Organization (WHO) and the Environment Protection Authority, along with numerous countries including Japan, have established regulatory limits for CN^−^. In Japan, the regulatory value of cyanide for drinking water is 0.01 mg/L, and the WHO drinking water guideline value is 0.07 mg/L [4]. Therefore, the development of a simple and efficient method for CN^−^ determination is highly desired.

Several instrumental analysis methods [5,6] based on the König reaction can be used to determine CN^−^, such as spectrophotometry [7,8,9] and flow injection methods [10,11,12]. However, these methods are expensive and time-consuming. Therefore, more convenient methods have been developed, including spot testing methods based on visual and colorimetric measurement using smartphones [13,14,15,16]. Additionally, methods based on fluorescence have also been reported [17,18], though these are not suitable for on-site analysis.

Unfortunately, on-site measurements using colorimetric methods are usually affected by environmental factors such as time and place. Therefore, colorimetric methods suitable for on-site measurements are still required. To this aim, recent progress in the miniaturization of analytical equipment has offered a unique platform to improve the equipment at a low cost using battery-powered equipment and three-dimensional (3D) printers [19,20,21,22,23,24,25].

**Scheme 1:**
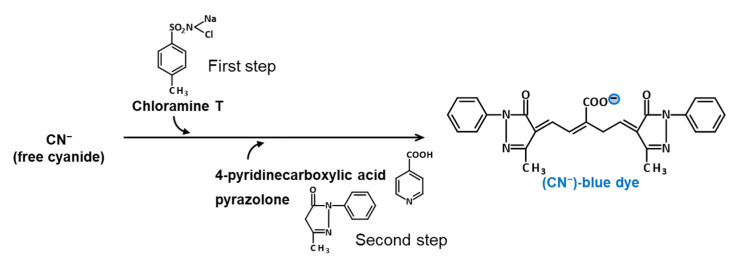
The colorimetric reaction of free cyanide to produce CN^−^-(blue dye).

In this study, we were interested in exploiting the simplicity of colorimetric methods to develop a rapid and simple method for on-site CN^−^ determination. As the measurement principle, we used dispersed particle extraction combined with solid-phase colorimetric analysis [26,27]. In this type of analysis, a colored target component is captured by an adsorbent via electrostatic adsorption or ion-pair extraction, and the concentration of the target component is determined according to the color of the fine particles that have naturally sedimented to the bottom of the microtube. In this method, a CN^−^-derived dye (hereinafter referred to as CN^−^-blue dye) produced by the reaction between CN^−^ and 4-pyridinecarboxylic acid [28] as shown in Scheme 1 was concentrated into microparticles via ion-pair extraction using Zephiramine as an ion pairing agent. To improve the reproducibility and suitability of the developed method for on-site measurement, we designed a mobile photography box (MPB) using a 3D printer to photograph the deposited particles with a smartphone and measure the color information (blue/(red + green + blue)). Furthermore, the method was adapted for the analysis of real samples, such as ground water and tap water.

## 2. Results and Discussion

### 2.1. Design of Equipment and Colorimetry for On-Site Analysis

**Figure 1:**
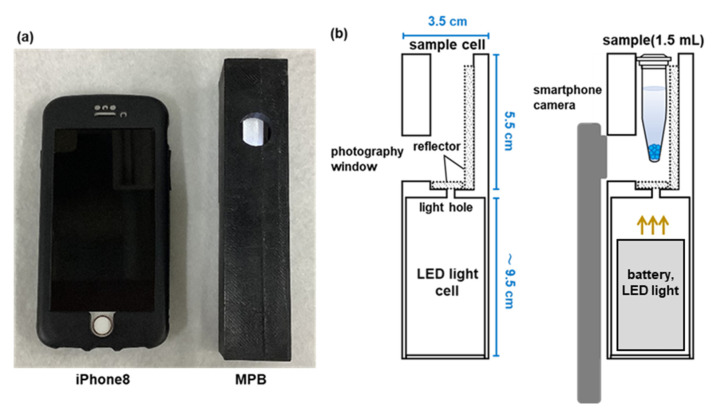
Outline of the mobile photography box (MPB). (**a**) Photograph of the actual product. (**b**) Schematic diagram. Yellow arrows indicate white light irradiation.

**Figure 2:**
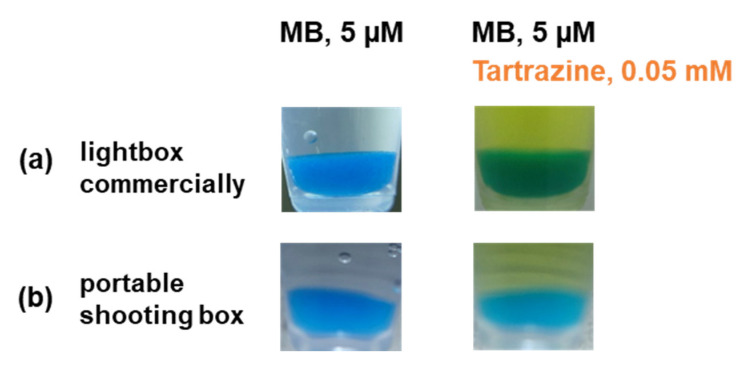
Illustrates the influence of the dissolved color component (yellow) on the photography of sedimented microparticles that have been colored by methylene blue (MB). The images were captured using (**a**) the commercially available shooting box and (**b**) the mobile photography box (MPB) fabricated for this study.

In image analysis using colorimetry, the shooting environment should be arranged to obtain accurate data. In addition, the colorimetric method must be sensitive and accurate. Therefore, in this study, a new MPB was fabricated using a 3D printer for on-site analysis (Figure 1). This device has a two-layer structure with a sample chamber on the top and a light source chamber on the bottom. A diffuser plate spray-coated in white was installed inside the sample chamber to uniformly diffuse the light source. The utilization of a white diffuser plate has been demonstrated to enhance the detection sensitivity of methylene blue (MB) by approximately 2.9 times in comparison to the use of a black diffuser plate. (Figure S1). With optimized light source intensity (Figure S2) and image measurement position (Figure S3), the MPB was designed so that the light source was illuminated from below. The average color value obtained from a wide area (200 × 200 pixels) in the vertical and horizontal directions was used. The measured values of MB obtained using the MPB were found to be equivalent to those obtained using a commercial shooting box. When colored tartrazine (yellow anionic dye) was added to the aqueous phase as a dissolved color component, the blue-colored microparticles were photographed with the commercially available shooting box as green due to the yellow solution (Figure 2a). Interestingly, when shooting with the MPB, it was possible to photograph the blue-colored particles without being affected by the yellow-colored solution (Figure 2b). This suggests that the interference of light due to the color of the solution can be reduced by irradiating from a certain direction (bottom) with an amount of light that can measure the scattering of the fine particles.

### 2.2. Development of a Simple Analysis Method for CN^−^

In this method, the efficiency of the recovery of the dye determines the final color tone of the particulates. Therefore, the optimal conditions, i.e., retention modes and particulate adsorbent, were screened to achieve a high dye recovery.

#### 2.2.1. Ion-Exchange Extraction Mode

**Figure 3:**
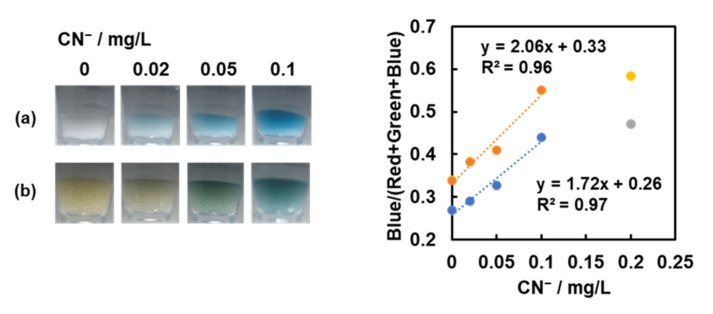
CN^−^-(blue dye) collection by ion exchange extraction mode using (a) DOWEX™ 1-X8 and (b) Wakogel^®^ 50SAXS ((SiO_2_)-R_3_NH_3_^+^). Adsorption conditions; CN^−^ concentration: 0.1 mg/L, amount of particles: 10 mg, and sample volume: 1 mL.

The charge of the CN^−^-(blue dye) is the most important parameter for constructing the solid-phase extraction system and optimizing the analytical conditions. The CN^−^-(blue dye) has been reported to have a negative charge (Scheme 1) in an acidic medium [28]. Therefore, in the present study, the application of the ion exchange extraction mode was initially investigated. Two types of anion exchangers, i.e., DOWEX™ 1-X8 (Fujifilm Wako Pure Chemical Industries, Ltd. (Osaka, Japan)) and silica-based Wakogel^®^ 50SAX (SiO_2_–R_3_NH_3_^+^), were examined. Figure 3a,b shows photos of the sediment obtained after extracting CN^−^-(blue dye) with 10 mg of DOWEX™ 1-X8 and SiO_2_–R_3_NH_3_^+^, respectively. The recovery ratios estimated by UV–Vis spectrophotometry analysis of the aqueous phases were 87% and 54%, respectively. Although DOWEX™ 1-X8 showed a higher recovery ratio than SiO_2_–R_3_NH_3_^+^, the yellow color of the anion exchanger material interfered with the determination of the concentration via visual and image colorimetry. Therefore, another extraction mode for the anion component was investigated.

#### 2.2.2. Ion-Pair Extraction Mode

Generally, an adsorbent that is compatible with the target component is selected. However, in this study, in order to improve the recovery rate, the composition of the target component was altered to utilize ion-pair adsorption, whereby ion-pairing agents that form pairs with the target components are added to generate ion pairs. In a previous work, we added sodium dodecyl sulfate, an anionic surfactant, as an ion-pairing agent to a Cr-(DPC)_3_ complex to generate an ion pair, which was extracted on a nonpolar XAD-7HP resin to improve the extraction efficiency [26,27].

**Figure 4:**
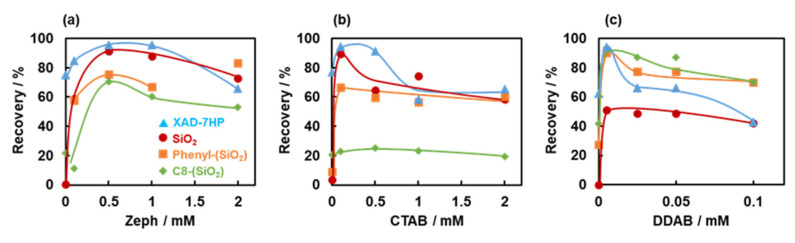
CN^−^-(blue dye) collection by ion-pair extraction mode using (**a**) Zeph, (**b**) CTAB, and (**c**) DDAB as an ion-pairing agent. Adsorption conditions; CN^−^ concentration: 0.1 mg/L, amount of particles: 10 mg, sample volume: 1 mL. The particle type (▲) is XAD-7HP, (◆) is C8-(SiO_2_), (■) is phenyl-(SiO_2_), and (●) is SiO_2_.

In the present study, three kinds of adsorbents, i.e., XAD-7HP and SiO_2_ modified with phenyl and octyl groups, and three ion pairing agents with different side chains (benzyldimethyltetradecylammonium chloride (Zeph), hexadecyltrimethylammonium bromide (CTAB), and didodecyldimethylammonium bromide (DDAB)) were examined (Figure 4). The trapping efficiency of the three adsorbents increased with increasing the ion pairing agent concentration and decreased above a particular concentration, which can be assigned as the critical micelle concentration (CMC) of the added ion pairing agents [29,30,31]. Above the CMC, the micelles formed in the aqueous phase compete with the ion pairs collected by the microparticles, decreasing the recovery efficiency. Silica gel is characterized by a negative charge in neutral solutions. However, when highly hydrophobic Zeph^+^ is adsorbed on the surface of silica gel, the surface is thought to become non-charged or even positively charged, depending on the concentration. Consequently, it is hypothesized that CN-(blue dye) is collected as an uncharged ion pair, Zeph^+^ • CN^−^(blue dye), or as an anion.

**Figure 5:**
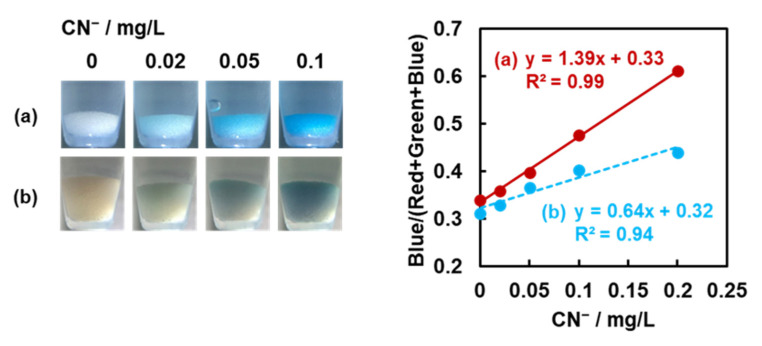
Comparison of microparticles for CN^−^-(blue dye) accumulation by ion-pair extraction mode. Adsorption conditions; ion pairing agent (Zeph) concentration: 0.5 mM, amount of particles: 10 mg, sample volume: 1 mL. Particle type (a) is SiO_2_ and (b) is XAD-7HP.

Figure 5 shows the photos and the values of color information (blue/(red + green + blue)) for XAD-7HP resin and SiO_2_ particles obtained by adding 0.5 mM Zeph as the ion pairing agent to several concentrations of CN^−^ solution. The addition of 0.5 mM Zeph as an ion-pairing agent resulted in a recovery rate of 90% or more (Figure 4a). Among the adsorbents evaluated, the XAD-7HP resin showed the highest recovery efficiency and was stable. However, when the XAD-7HP resin was photographed with the portable shooting box to measure the color information (blue/(red + green + blue)), the sensitivity was low, which can be attributed to light transmittance due to the adsorbent (Figure 5). Therefore, SiO_2_-based adsorbents having high permeability are suitable. In our previous work, to optimize the adsorbent, SiO_2_ was coated with a cationic surfactant to change the surface state of SiO_2_ and increase the extraction efficiency [27]. In this investigation, it seems that the CN^−^-(blue dye) ion pair was accumulated on SiO_2_ by adding an excess amount of cationic surfactant as an ion-pairing agent. The use of SiO_2_ as the adsorbent resulted in a sensitivity of the color information (blue/(red + green + blue)) regarding CN^−^-(blue dye) extraction that was more than twice as high as that observed when XAD-7HP was used. Accordingly, SiO_2_ was selected as the adsorbent for its excellent recovery efficiency and sensitivity.

#### 2.2.3. Color Development Speed and Measurement Time

A stable coloration of CN^−^ is generally required for about 30 min. In addition, the coloring reagent has a low solubility in water. Although this problem is usually overcome by dissolving the coloring reagent in heated water or an organic solvent before use, the use of heating or organic solvents is not convenient for on-site analysis. Therefore, only water without heating was used in this study. The measurement time was determined because it is also affected by the color development time of the target component. As the CN^−^-(blue dye) was collected by ion-pair extraction, the effect of the addition of ion-pairing agents on the coloring time was examined.

**Figure 6:**
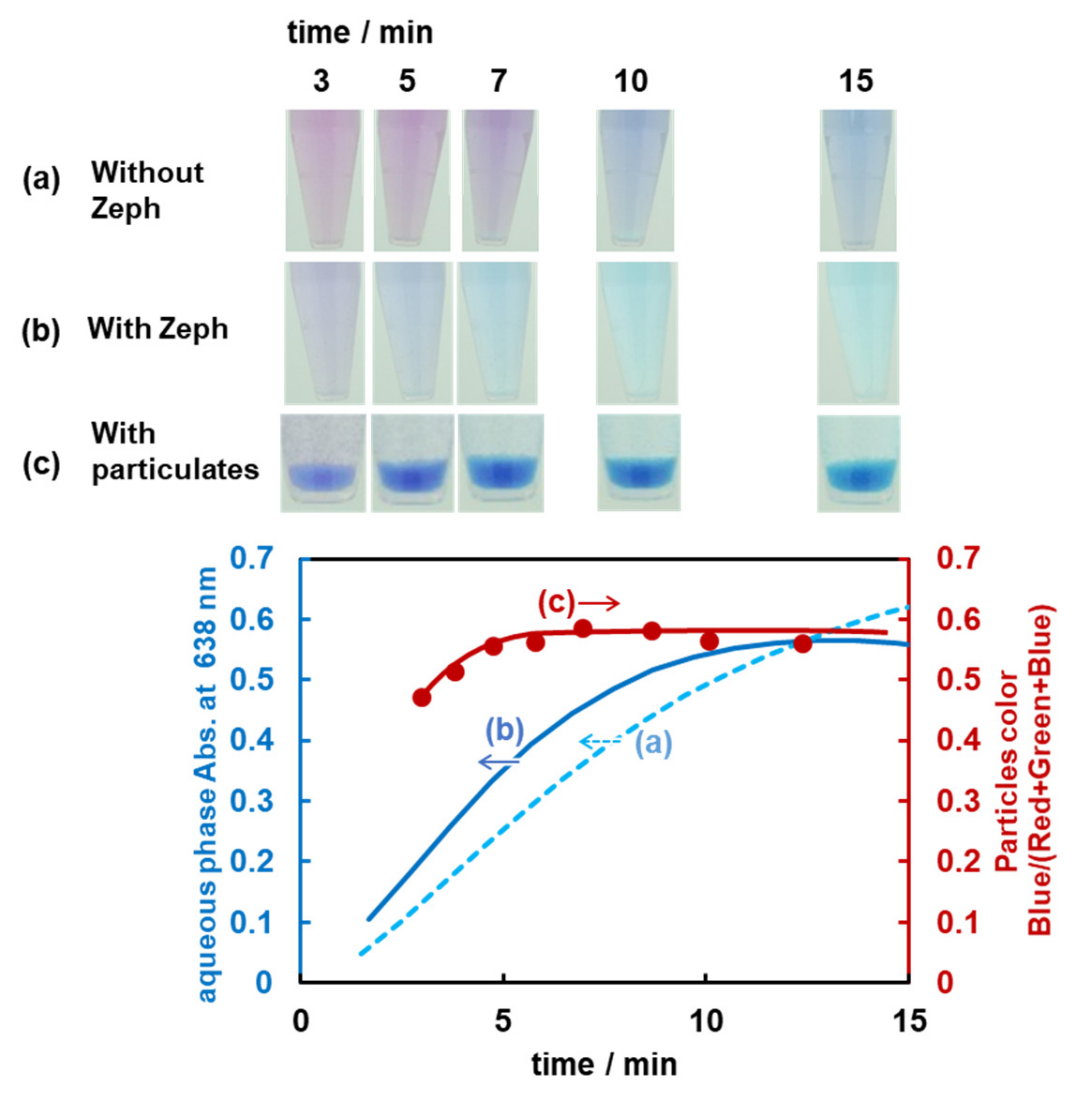
The investigation of the coloring reaction time for the reaction with 4-pyridinecarboxylate tetrahydrate and 3-methyl-1-phenyl-5-pyrazolone (second step) (a) without Zeph (0.5 mM) and particles, (b) with Zeph (0.5 mM) and without particles, and (c) with Zeph (0.5 mM) and particles. Adsorption conditions; CN^−^ concentration: 0.5 mg/L, particles: 10 mg SiO_2_, sample volume: 1 mL. The colorimetric determination of solution was performed by measuring absorbance at 638 nm.

The time dependence of the coloring reaction at 0.5 mg/L CN^−^ was measured according to the absorbance of the solution and an image analysis of the adsorbent deposit. Figure 6 shows a photograph of the color change with time. When 0.5 mM Zeph was not added as an ion-pairing agent (blue dotted line in Figure 6), the reaction proceeded slowly, and the color was not stable after 15 min. In contrast, in the presence of 0.5 mM Zeph (blue line), the coloration was observed after 10 min and became constant faster. Although the specific effect of the surfactant on this color reaction is not known, it seems likely that adding a surfactant at a concentration higher than the CMC improves the dispensability of the reagent by facilitating its dissolution on the micelles. When 10 mg of SiO_2_ was added as an adsorbent (red line), the coloration was faster than the reaction in the aqueous phase and stabilized after 7 min. Under the optimized conditions, it is possible to measure CN^−^ was performed within 10 min in this technique.

### 2.3. Optimized Procedure for the Colorimetric Determination of CN^−^

After optimizing the conditions for the extraction and measurement of CN^−^, the method was established as follows: the necessary adsorbent particulates and reagents were packed into a microtube in powder form before injection of the sample. First step: 1 mL of the sample containing 0.62 mg of chloramine T, NaH_2_PO_4_ (33 mg) and Na_2_HPO_4_ (35 mg) was added to the microtube. The addition of 1 mL of water sample results a phosphate buffer solution with pH 7.2. Second step: 300 mg of 4-pyridinecarboxylate tetrahydrate, 3 mg of 3-methyl-1-phenyl-5-pyrazolone, 0.2 mg of Zeph (0.5 mM), and 10 mg of SiO_2_ particulates were added to the microtube. The microtube was shaken for 30 s and held for 7 min. The CN^−^ concentration was determined as described in the next section.

### 2.4. Calibration Curve for CN^−^

**Figure 7:**
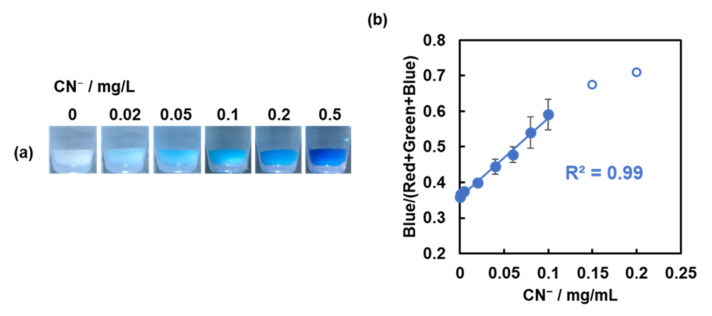
The determination of CN^−^. (**a**) The photographs of a microtube for analyzing different concentrations of CN^−^ range between 0 and 0.5 mg/L. (**b**) The calibration curve obtained from the image analysis of the deposited microparticles that had been concentrated CN^−^-(blue dye) by ion-pair extraction. Adsorption conditions; ion pairing agent (Zeph) concentration: 0.5 mM, particles: 10 mg SiO_2_, sample volume: 1 mL.

Figure 7a shows the photographs of a microtube containing a CN^−^ concentration of 0–0.5 mg/L taken with the MPB. The image analysis was conducted on the deposited microparticles that had been concentrated CN^−^-(blue dye) by ion-pair extraction. The changes in the CN^−^-(blue dye) according to the CN^−^ concentration were determined as the ratio between each color tone value and the sum of the obtained color tone values, with the blue/(red + green + blue) value showing the most sensitive change. As shown in Figure 3a, it was possible to judge by the visual colorimetric method at 0.02–0.5 mg/L. The calibration curve of y = 2.24x + 0.36 (R^2^ = 0.99) was obtained at 0.005–0.100 mg/L using the results of the image colorimetric analysis (Figure 7b). This method showed a detection limit of 5 µg/L (3σ_B_) and could measure CN^−^ at concentrations lower than the tap water quality standard (not detected <0.01 mg/L).

### 2.5. Effect of Interferences

To determine the effect of interferences on the recovery rate of CN^−^, several substances that usually coexist with CN^−^ in industrial wastewater were added and the analysis was conducted using the developed method (Table 1). The error was calculated using the standard deviation of the results of three repeated measurements. Since CN^−^ tends to form complexes with metals, even small amounts of Ag^+^ and Co^2+^ affected the color reaction and hindered the measurement. In contrast, good recovery rates were obtained in the presence of Zn^2+^ and Cr^3+^ even at high concentrations. Meanwhile, the presence of anions exerted little effect; the competition with ion pairing agents was small, and sodium citrate was measured by diluting. The complexed product can be measured as a total cyanide compound by incorporating a distillation operation as a pretreatment.

### 2.6. Application to Real Samples

The method was validated using groundwater and tap water as shown in Table 2. The standard deviation of the results of three repeated measurements was employed in the calculation of the error. In both cases, a recovery rate of about 90% was obtained compared with distilled water, and the measurement errors were 4.4% and 13.0%, respectively.

## 3. Materials and Methods

### 3.1. Chemicals and Materials

Potassium cyanide, sodium 4-pyridinecarboxylate tetrahydrate, 3-methyl-1-phenyl-5-pyrazolone, NaH_2_PO_4_, Na_2_HPO_4_, HCl, NaOH, and CTAB were provided by Fujifilm Wako Pure Chemical Industries, Ltd. (Osaka, Japan). Chloramine T trihydrate and DDAB were purchased from Sigma Aldrich, Ltd. (St. Louis, MO, USA). Zeph was provided by DOJINDO LABORATORIES, Ltd. (Kumamoto, Japan). All reagents were of analytical grade and used as received without further purification. Milli-Q water was used for the preparation of the solutions.

Wakogel^®^ 50SAX (trimethylaminopropyl), Wakogel^®^ 50C8 (octyl), Wakogel^®^ 50Phenyl (40–63 µm), and Wakogel^®^ C-300 (45–75 µm) sedimentable fine particle adsorbents for column chromatography and DOWEX™ 1-X8 (Cl^−^) (200–400 mesh) anion-exchange resin were purchased from Fujifilm Wako Pure Chemical Industries, Ltd. (Osaka, Japan). Amberlite^®^ XAD-7HP (20–60 mesh) was provided by Sigma Aldrich, Ltd. (St. Louis, MO, USA) and ground in a mortar, and the fraction with a size of 0.36 ± 0.10 mm was separated via sedimentation in water.

### 3.2. Apparatus

An ultraviolet–visible (UV–Vis) spectrophotometer (Shimadzu, Kyoto, Japan, UV-1800) was used for the colorimetric analysis. Images were taken using an EOS KISS X7 EF-S18-55 mm F3.5-5.6 IS STM (Canon, Tokyo, Japan) digital camera or an iPhone 8 in a lightroom (G • T • B • T, HAD QT-310, color temperature 5400 K). A 3D printer XYZ printing PRO and a 3D printer filament for da Vinci ABS black was used to create the portable shooting box that can be attached to a mobile phone.

### 3.3. Image Analysis

Photographs of the particulates in a microtube were taken in the designed MPB using an iPhone 8. The photographs were transferred to a personal computer and converted to an 8-bit color scale using the ImageJ 1.53 software. The red, green, blue, and gray strength values of the adsorbents precipitated in the microtube were obtained using an area of 200 × 200 pixels at the center of the microtube, and the average value of three measurements is provided.

## 4. Conclusions

The newly developed colorimetric method allows direct measurement of the color of the target component collected on particles by illuminating from below and capturing the image from a perpendicular direction. This method has several advantages:

1. Excellent portability as it is battery powered.
2. High sensitivity and repeatability.
3. Less susceptibility to interference from other coloring components.

This method has been successfully applied to the simple and highly sensitive analysis of free cyanide. The results showed that cyanide could be detected with high sensitivity (detection limit: 0.005 mg/L) in a much shorter time, i.e., within 10 min, than the conventional absorbance spectrophotometric method, which typically takes 30 min. This newly developed method is therefore highly effective for the rapid and sensitive detection of free cyanide. The present study demonstrates the successful application of the proposed method to groundwater and tap water.

This portable and sensitive colorimetric method holds promise for broader on-site applications beyond cyanide detection, potentially extending to other environmental and industrial analyses. Future improvements could focus on improving smartphone integration, automating image analysis, and increasing versatility for real-time monitoring. Such advances will help meet the growing demand for rapid and reliable field testing.

## Figures and Tables

**Table 1:** Effect on measurement when added coexisting components.

Ion	*n* Count	Added/mg/L	Recovery/%
none (CN^−^/0.05 mg/L)	5	-	100.0 ± 20.0
Ag^+^	3	5	n.d.
Co^2+^	3	10	n.d.
Cu^2+^	3	30	9.6 ± 19.9
Zn^2+^	3	100	89.0 ± 12.5
Cr^3+^	3	100	96.5 ± 3.0
NO_3_^−^	3	600	91.6 ± 21.5
SO_4_^2−^	3	15	91.6 ± 21.5
sodium citrate	3	0.04 M	22.2 ± 2.8
glucose	3	1000	91.6 ± 21.5

n.d. = no detection.

**Table 2:** Addition recovery of sample water.

Sample	*n* Count	Residual Chlorine/mg/L	Added CN^−^/mg/L	Recovery/%
groundwater	3	0.2	0.05	93.3 ± 4.4
tap water	3	0.4	0.05	91.7 ± 13.0

## Data Availability

Data are contained within the article.

## Supplementary Materials

The following supporting information can be downloaded at: https://www.mdpi.com/article/10.3390/molecules29225371/s1. Figure S1: The effect of the diffuser plate installed in the mobile photography box (MPB) on the color measurement of microparticles; Figure S2: Optimization of light source intensity; Figure S3: Optimization of light source intensity.
