# Supplementary material for: On-Site Sensitive Colorimetry for Free Cyanide by Using Ion-Pair Solid-Phase Extraction with Sedimentable Dispersed Particulates and Mobile Photography Box
Source: Molecules. 2024 Nov 14;29(22):5371. doi: 10.3390/molecules29225371 (PMC11596628; doi:10.3390/molecules29225371)
Supplement: Supplementary file 1 [file molecules-29-05371-s001.zip › molecules-3313911-supplementary.pdf]

molecules

MDPI

Contents

Figure S1. The effect of the diffuser plate installed in the mobile photography box (MPB) on the color measurement of microparticles.

Figure S2. Optimization of light source intensity.

Figure S3. Optimization of light source intensity.

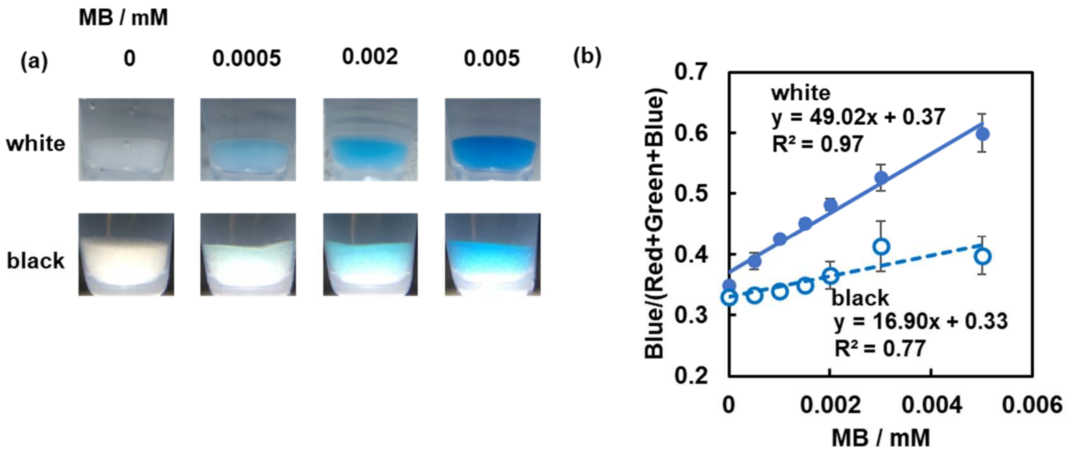

Figure S1. The effect of the diffuser plate installed in the mobile photography box (MPB) on the color measurement of microparticles. (a) Photographs of microparticles adsorbed with different concentrations of methylene blue (MB) using white or black diffuser. (b) The calibration curves obtained from the image analysis of the deposited microparticles that had been accumulated MB. Adsorption conditions; particles: 10 mg of silica gel, sample volume: 1 mL. Colorimetry conditions; ImageJ 200×200 pixel, camera: iPhone 8.

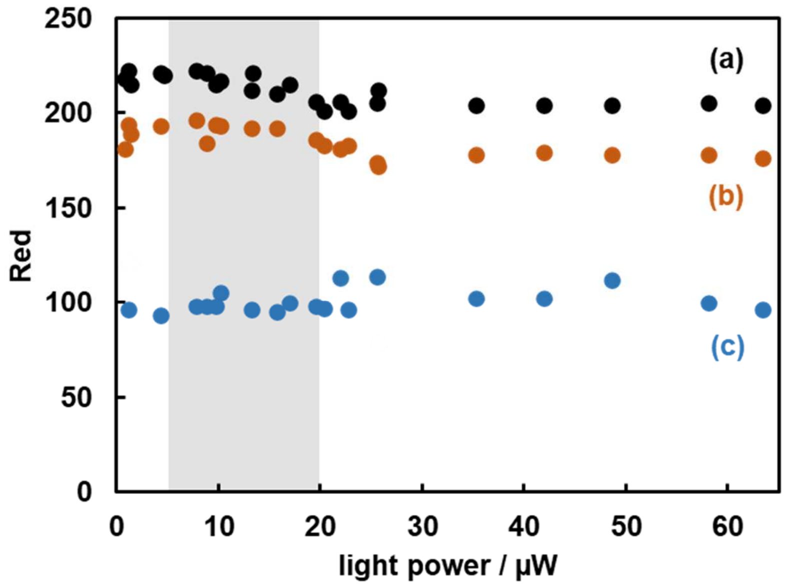

Figure S2. Optimization of light source intensity. The red values obtained from photographs of microparticles adsorbed with different concentration of methylene blue (MB) under different light source intensities. MB concentration (a) 0 μM, (b) 0.2 μM, (c) 1 μM. Adsorption conditions; particles: 10 mg of silica gel, sample volume: 1 mL.
Colorimetry conditions; ImageJ 200×200 pixel, camera: iPhone 8. Gray line is used buttery LED light.

Measurements were made at different light intensities and the R values obtained from the microparticles adsorbed a different concentration of MB. The dry-cell LED light source can be used as a light source because the light intensity (grey area) is stable.

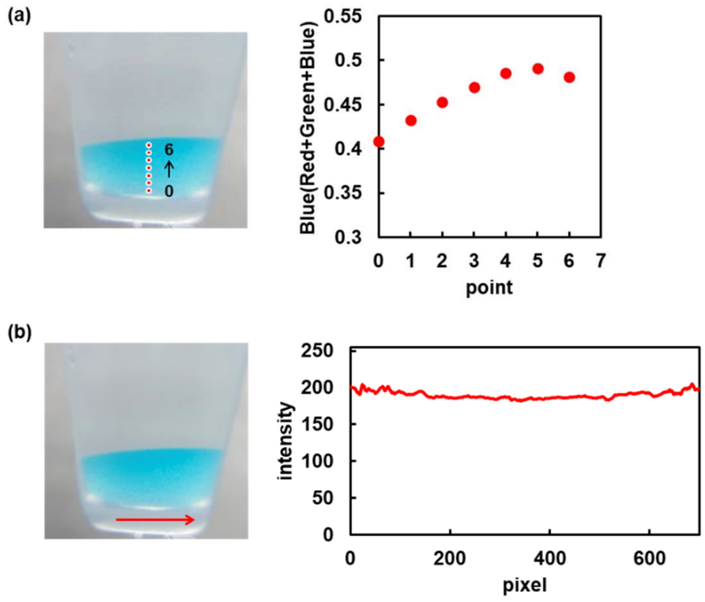

Figure S3. Optimization of image measurement position. (a) Measurement results for each point in the vertical direction, (b) measurement results for each point in the parallel direction. Adsorption conditions; particles: 10 mg of silica gel, sample volume: 1 mL. Colorimetry conditions; ImageJ 200×200 pixel, camera: iPhone 8.

The mobile photography box (MPB) is designed that the light source is illuminated from below. As a result, there is a gradient in color values because of the direction of the light source. Therefore, the average value obtained from a wide area (200 x 200 pixels) in the vertical and horizontal directions was used.
